# Statutes of the International Committee on Systematics of Prokaryotes – July 2025 revision

**DOI:** 10.1099/ijsem.0.006918

**Published:** 2025-09-30

**Authors:** David R. Arahal, Markus Göker, Richard L. Hahnke, Célia M. Manaia, Edward R.B. Moore, Aharon Oren, Iain C. Sutcliffe, Stefano Ventura

**Affiliations:** 1 Departamento de Microbiología y Ecología, Universitat de València, 46100 Burjassot (Valencia), Spain; 2 Leibniz Institute DSMZ – German Collection of Microorganisms and Cell Cultures, Department of Bioinformatics and Databases, Inhoffenstrasse 7B, 38124 Braunschweig, Germany; 3 Leibniz Institute DSMZ – German Collection of Microorganisms and Cell Cultures, Department of Microorganisms, Inhoffenstrasse 7B, 38124 Braunschweig, Germany; 4 Universidade Católica Portuguesa, CBQF – Centro de Biotecnologia e Química Fina – Laboratório Associado, Escola Superior de Biotecnologia, Rua Diogo Botelho 1327, 4169-005 Porto, Portugal; 5 Department of Infectious Disease and Culture Collection University of Gothenburg (CCUG), Institute for Biomedicine, Sahlgrenska Academy, University of Gothenburg, Sweden, P.O. Box 7193, SE-402 34, Gothenburg; 6 Department of Plant and Environmental Sciences, The Institute of Life Sciences, The Hebrew University of Jerusalem, Edmond J. Safra Campus, Jerusalem 9190401, Israel; 7 Northumbria University, Newcastle Upon Tyne NE1 8ST, UK; 8 IRET-CNR, Research Institute on Terrestrial Ecosystems, National Research Council, Via Madonna del Piano 10, 50019 Sesto Fiorentino, Italy; 9 National Biodiversity Future Center (NBFC), Palermo, Italy

**Keywords:** International Committee on Systematics of Prokaryotes (ICSP), statutes

## Abstract

The members of the International Committee on Systematics of Prokaryotes (ICSP) have recently voted on a proposed revision of its statutes. We here present the outcome of the vote and the text of the revised statutes, as approved by the ICSP.

## Introduction

The International Committee on Systematics of Prokaryotes (ICSP) serves to administer the rules of prokaryotic nomenclature via the International Code of Nomenclature of Prokaryotes (ICNP) [1], ensures the publication of the *International Journal of Systematic and Evolutionary Microbiology* and works to represent the interests of the microbiological disciplines regarding prokaryotic nomenclature. The functions and mechanisms of operation of the ICSP are defined in its Statutes, which were last revised in 2019 [2].

A new revision of the statutes was proposed in March 2024 [3]. Major changes proposed included: (1) giving IUMS Members Societies the right to appoint more than one Full member to the ICSP, depending on the size of the membership; (2) giving Life Members the same voting rights as Co-opted Members; (3) separation of powers between the legislative and judiciary branches; (4) formal regulation of the work of Ad Hoc Subcommittees in analogy to the work of Subcommittees on Taxonomy; (5) clarification and streamlining of debates, revisions and voting procedures; and (6) extending the time that Committee members can serve on the Executive Board and broaden the range of members who can serve as Members-at-Large.

To comply with Article 13(b)(4) and Article 4(d) of the Statutes, a public discussion of the proposal was open between 5 March and 4 September 2024. The authors of the proposed revisions further discussed the comments raised and made a number of minor technical corrections that did not affect the content of the document.

The Full Members and Co-opted Members of the ICSP voted on the proposal between 25 January and 25 July 2025. The ballot form and content, including a summary of the public discussion, are found online at https://doi.org/10.5281/zenodo.16125331. Members could vote on all proposed amendments collectively or vote separately on blocks of amendments covering different topics. H. Christensen (Vice-Chair of the ICSP Judicial Commission) and P. Nielsen (ICSP Treasurer) served as the tellers.

Of the 55 ICSP members eligible to vote, 48 returned the ballot form. The result was as follows:

**Table:** 

	Endorse the proposed version	Retain the original version	Abstention
Block E – giving IUMS Member Societies the right to appoint more than one Full member to the ICSP, depending on their size	39	7	2
Block L – giving Life Members the same voting rights as Co-opted Members	42	6	0
Block M – miscellaneous clarifications, adaptations to modern practice, filling of gaps and other improvements of the wording	44	3	1
Block P – separation of powers between the legislative and judiciary branch	46	1	1
Block U – formal regulation of the work of Ad Hoc Subcommittees in analogy to the work of Subcommittees on Taxonomy	46	2	0
Block V – clarification and streamlining of debates, revisions and voting procedures	44	2	2
Block X – extending the time that committee members can serve on the Executive Board and broaden the range of members who can serve as Members-at-Large	41	6	1

Based on the outcome of the vote, the following revision of the statutes was approved, to take effect with the publication of this document.

## Statutes of the International Committee on Systematics of Prokaryotes, its subcommittees and the Judicial Commission on Prokaryote Nomenclature of the Bacteriology and Applied Microbiology Division of the IUMS

### Article 1

**Name.** The name of the Committee shall be the International Committee on Systematics of Prokaryotes of the Bacteriology and Applied Microbiology (BAM) Division of the International Union of Microbiological Societies (IUMS). For the purpose of abbreviation in all official documents, it will be referred to as the ICSP.

### Article 2

Membership on the ICSP shall be **Full Members, Co-opted Members** and **Life Members**. Each Member Society (as defined in Article 3 of the constitution of the BAM Division of IUMS) of the BAM Division of the IUMS is entitled to appoint at least one delegate to act as a Full Member to the Committee. The ICSP may co-opt other members and appoint Life Members.

(a) **Full Members.** Each Member Society of the BAM Division of the IUMS is entitled to appoint at least one Full Member to the ICSP by means of a letter to the Executive Secretary of the ICSP. Full Members may be appointed at any time. A Member Society is not obligated to appoint a member, and such positions will be considered to be vacant until a member is appointed.
  (1) The tenure of a Full Member is for one 3-year term. A term begins upon appointment by a Member Society and ends either upon appointment of another Full Member by the Member Society or on 1 April 2020, or every 3 years thereafter. Full Members shall be eligible for reappointment by their Member Societies for an indefinite period.
  (2) When a Society ceases to be a member of the BAM Division of the IUMS, its appointed delegate(s) shall continue to be Full Members until the end of the term.
  (3) The number of Full Members that can be appointed as delegates to the ICSP by a Member Society of the BAM Division of the IUMS is dependent upon the number of members of the Member Society as follows: default, 1 delegate; 1–499 members, 1 delegate; 500–999 members, 2 delegates; and >1,000 members, 3 delegates.
  (4) For the purpose of appointing more than one delegate, a Member Society of the BAM Division of the IUMS must provide evidence for the current number of its own members to the Executive Secretary of the ICSP prior to the appointment.
  (5) If the change in number of members of a Member Society affects the number of appointed delegates, all appointed delegates shall continue to be Full Members until the end of the term.
(b) **Co-opted Members.** The ICSP may co-opt members for the purpose of assisting with the work of the Committee.
  (1) Proposals for co-opted membership must be submitted to the Executive Secretary of the ICSP. Proposals must include a letter of nomination giving the case for co-option. The letter of nomination can be issued by any party, including self-nominations. All proposals must be supported in written form by at least two Full Members. Candidates must be approved by a vote of the Full Members [see Article 2(e)].
  (2) The tenure of a Co-opted Member is for one 3-year term. A term begins upon appointment and ends on 1 May 2026, or every 3 years thereafter. If Co-opted Members wish to renew their membership, they should notify the Executive Secretary of the ICSP within 2 months before the start of a new term, so that a ballot can be arranged, and provide a brief justification for renewal.
  (3) Co-opted Members have the right to vote in all matters except the election of officers, Co-opted Members and Life Members and emendation of the ICSP Statutes.
(c) **Life Members.** The ICSP may appoint to Life Membership of the ICSP as many as 5 retired individuals who have rendered distinguished service to the ICSP. Life Members may not be the delegates of any Member Society.
  (1) Proposals for Life Membership must be submitted by at least two Full Members to the Executive Secretary of the ICSP with an appropriate justification and approved by a vote of the Full Members [see Article 2(e)].
  (2) Life Members have the right to vote in all ICSP matters except the election of officers, Co-opted Members and Life Members and emendation of the ICSP Statutes. Life Members are not eligible for election to office and membership in the Executive Board.
(d) **Recognition of Alternates.** If a Full Member of the ICSP cannot attend a meeting of the ICSP, an Alternate having all the rights of a Full Member, except that of eligibility to office in the ICSP, will be appointed in accordance with the following provisions.
  (1) A Full Member of the ICSP who cannot attend the meeting shall have the right to appoint an Alternate from within the Full Member’s own Member Society or, if this is not possible, from within any other Member Society of the BAM Division of the IUMS.
  (2) Notice of appointment of Alternates shall be made to the Executive Secretary of the ICSP prior to the meeting.
(e) **Voting.** Members are entitled to one vote of the Committee. Decisions require a vote by a majority (i.e. more than 50%) of the members eligible to vote and shall be reached on the basis of a majority of those members who vote. The decision shall be based on the votes received within the allotted time period. All ballots should have abstention as an option. The standard voting period is 6 weeks.

### Article 3

#### Functions of the ICSP

(a) To represent the diversity of interests of different microbiological disciplines on matters concerning the nomenclature of prokaryotes.
(b) To study, edit and disseminate the *International Code of Nomenclature of Prokaryotes* (ICNP). Interpretations of the Code that underlie the preparation and publication of Opinions are a task assigned by the ICSP to its Judicial Commission (see Article 8).
  (1) To publish the ICNP (see Article 13).
(c) To establish publications deemed necessary for advancement of the systematics of prokaryotes.
  (1) To maintain and disseminate the ‘Validation Lists’ and the *Approved Lists of Bacterial Names* (see Article 9).
  (2) To oversee publication of the *International Journal of Systematic and Evolutionary Microbiology* (IJSEM) (see Article 14).
(d) To elect members of the Judicial Commission as vacancies occur and to replace the members of the several classes as their terms expire.
(e) To consider and vote upon all recommendations communicated by the Editor-in-Chief of the ICNP for emendation of the ICNP (see Article 13).
(f) To receive reports of the Subcommittees on Taxonomy and ensure their dissemination (see Article 6).
(g) To organize and sponsor regional, national or international conferences on the systematics of prokaryotes, either independently or in conjunction with meetings organized by other professional societies. Conferences may be held electronically and made available via the Internet or other means as technology advances (see Article 4).
(h) To elect the Executive Board (EB) of the ICSP and to authorize the EB-ICSP to perform such functions as are defined in the statutes and such other functions as the ICSP may determine (see Article 5).
(i) To vote on nominations of Co-opted Members of the ICSP [see Article 2(b)].
(j) To appoint as Life Members of the ICSP individuals who have rendered distinguished service to the ICSP [see Article 2(c)].
(k) To take other actions that ensure the proper application of the Code.

### Article 4

#### Conduct of business by the ICSP

(a) The business of the ICSP will be conducted at Plenary Meetings or electronic forums. Plenary Meetings will be held at such times and places as may be determined by the EB-ICSP. When possible, Plenary Meetings shall be held at intervals of not more than 3 years and shall be held in association with other meetings, conferences or congresses sponsored by the ICSP, IUMS or other scientific societies.
(b) All committees, subcommittees and commissions of the ICSP have the authority to conduct business by electronic media.
(c) When conducting the business of the ICSP in electronic media, the format of the meeting is chosen by the Executive Board. Generally, time should be allowed for sharing the relevant materials on the topic under discussion, deliberations by the members, and voting.
(d) The business of the ICSP will be conducted publicly, with minutes reported of all meetings of the ICSP and its committees. Minutes of the Plenary Meeting will be reported by publication in the IJSEM. Minutes of the EB-ICSP, Judicial Commission, Subcommittees, editorial boards and other committees will be made publicly available by appropriate means, possibly including posting on a permanently publicly accessible website or repository, as determined by the committees.

### Article 5

**The Executive Board-ICSP**. The membership of the Executive Board (EB) shall be: Chair and Vice-Chair, Executive Secretary, Secretary for Subcommittees on Taxonomy, Treasurer and two Members-at-Large. The Chair, Vice-Chair, and Secretary of the Judicial Commission shall be ex officio non-voting members of the Executive Board. No single member may hold more than one function or cast more than one vote. No member of the EB-ICSP may serve on the Executive Board in any capacity for more than three consecutive terms, or in the same capacity for more than two consecutive terms. After an absence of one term, former members of the EB-ICSP who have served three consecutive terms shall be eligible for re-election as an officer on the Executive Board.

(a) **Election of the Executive Board.** The terms of office for members of the EB-ICSP begin on 1 September 2020 and every 3 years thereafter. One term is for 3 years. Members of the EB who are Full Members or Co-opted Members at the beginning of their term will retain their membership status until the end of their term, even if their Member Society appoints another delegate for the next term or the co-option is not renewed. The Chair and Vice-Chair will be elected from the Full Members. The Executive Secretary, the Secretary for Subcommittees on Taxonomy, the Treasurer and two Members-at-Large will be elected from the Full and Co-opted Members.
(b) **Functions of the EB-ICSP**
  (1) To organize meetings and electronic forums of the ICSP plenary [see Article 4(a)].
  (2) To conduct the business of the ICSP between plenary meetings as directed by the ICSP [see Article 4(b)].
  (3) To organize regional, national and international conferences and symposia on the systematics of prokaryotes or other appropriate topics.
  (4) To appoint Subcommittees (see Article 6), either on its own initiative or at the request of others, and to facilitate meetings of the Subcommittees at the plenary meetings of the ICSP or elsewhere.
  (5) To provide for the initial appointment and subsequent election of Chairs and Secretaries of Subcommittees on Taxonomy (see Article 6).
  (6) To deal with such business as the Judicial Commission may request (see Article 7).
  (7) To recommend to the ICSP the establishment of any Committees that are deemed necessary for the work and functioning of the ICSP and to consider recommendations submitted to it by those committees.
  (8) To appoint or replace individuals to implement and keep up-to-date the representation of the ICSP on electronic media, such as websites or social media, as deemed appropriate for the purposes of the ICSP.
  (9) Upon approval of the ICSP to:
    (a) establish publications authorized by the ICSP (see Articles 9 and 14).
    (b) negotiate such contracts as may be necessary for the issuance of publications authorized by the ICSP.
    (c) establish such Trusts or enter into such agreements as may be advisable for the auditing and administration of funds that may be designated for the payment of the necessary operating expenses of the ICSP and its subordinate agencies, whether such funds originate from grants, gifts, royalties, the sale of publications or other sources.
    (d) request from appropriate agencies grants for the necessary expenses of the ICSP and its subordinate agencie
  (10) To ensure the proper functioning of the organizations and officers of the ICSP.
  (11) To perform such other functions enumerated in the Sstatutes or assigned to it by the ICSP.
(c) **Duties of the Chair and Vice-Chair of the ICSP.** In addition to the duties specified below, the Chair and Vice-Chair shall be ex officio, but non-voting, members of all Committees, Commissions and Subcommittees of the ICSP, unless otherwise stated.
  (1) Duties of the Chair of the ICSP are:
    (a) To preside at meetings of the ICSP and its Executive Board (see Article 4).
    (b) With the other members of the Executive Board, to prepare the agenda for meetings of the EB-ICSP [see Article 4(d)].
    (c) With the other members of the Executive Board, to prepare the agenda for meetings and forums of the ICSP [see Article 4(a)].
    (d) To serve on the Publications Committee (see Article 9).
    (e) To serve on the Editorial Board for the ICNP [(see Article 13).
    (f) To assume such duties as may be requested by the ICSP or determined by the functions of the EB-ICSP.
  (2) Duties of the Vice-Chair are:
    (a) To preside at the meetings of the ICSP and EB-ICSP in the absence of the Chair [see Article 4(d)].
    (b) With the other members of the Executive Board, to prepare the agenda for the meetings and forums of the ICSP [see Article 4(a)].
    (c) To assume such duties as may be requested by the ICSP or determined by the functions of the EB-ICSP.
    (d) To chair the Publications Committee (see Article 9).
    (e) To serve on the Editorial Boards for the ICNP and IJSEM (see Articles 13 and 14).
(d) **Duties of the Executive Secretary.** In addition to the duties specified below, the Executive Secretary shall be responsible to the Executive Board in all matters associated with communications between the ICSP and the Executive Board of the IUMS, Division Council of BAM and Member Societies. The Executive Secretary shall perform the following duties:
  (1) Be Secretary of the EB-ICSP and the ICSP and submit the minutes of the plenary meetings for publication in the IJSEM [see Article 4(a)].
  (2) Be secretary to the electronic meetings of the EB-ICSP and ICSP and report the minutes in an appropriate manner, as determined by the plenary of the ICSP or, in the absence of specific instructions, by the EB-ICSP [see Article 4(d)].
  (3) With the other members of the EB-ICSP, to prepare the agenda for meetings of the EB-ICSP [see Article 4(d)].
  (4) Prepare, in cooperation with other members of the EB-ICSP, the agenda for meetings and forums of the ICSP [see Article 4(a)].
  (5) Request nominations from Member Societies for Full Members to the ICSP. Receive nominations for appointment of Full, Co-opted and Life Members and transmit these to the EB-ICSP.
  (6) Receive nominations for Alternates for meetings of the ICSP in accordance with Article 2(d).
  (7) Transmit to the ICSP such recommendations from the Judicial Commission as may require action by the ICSP [see Article 7(a)].
  (8) If the members are asked to vote upon any proposal, to tabulate and announce the result of the ballot and to certify the result to the Chair of the ICSP and to the Chair of the Judicial Commission.
  (9) Prepare every 3 years a complete list of all members and officers of the ICSP and the Judicial Commission and forward these to the Secretary Treasurer of the BAM Division of IUMS and the Chair of the ICSP.
  (10) Present every 3 years a report covering all pertinent actions of the ICSP and the Judicial Commission and other committees of the ICSP to the plenary of the ICSP and the Secretary of the BAM Division of the IUMS.
  (11) To maintain an archive of the important documents of the ICSP, including names and affiliations of its members and officers, minutes of meetings, copies of contracts, correspondence regarding any prizes awarded by the ICSP, reports of the treasurer and documents related to Ad Hoc Subcommittees.
  (12) Perform such duties as the EB-ICSP may from time to time determine.
(e) **Duties of the Treasurer.** The Treasurer shall receive funds which may be made available to the ICSP from any source and distribute them as directed by the EB-ICSP or its Chair.
  (1) Checks or requests for electronic fund transfers issued by the treasurer shall bear the Treasurer’s signature but must have been authorized in writing by the Chair of the ICSP or the Vice-Chair of the ICSP.
  (2) A statement of accounts shall be furnished by the Treasurer to the EB-ICSP before 1 September of each year. In addition, a statement will be furnished to the ICSP at each Plenary Meeting of the ICSP and to the Secretary Treasurer of the BAM Division of the IUMS as requested.
(f) **Duties of the Secretary for Subcommittees on Taxonomy.** The Secretary for Subcommittees on Taxonomy shall be responsible to the Executive Board for all matters associated with the Subcommittees of the ICSP. The Secretary for Subcommittees on Taxonomy shall perform the following duties:
  (1) Serve as a non-voting member of each Subcommittee (see Article 6).
  (2) Act as a liaison between the officers of the Subcommittees and the ICSP, transmitting requests and recommendations to the EB-ICSP and/or Judicial Commission, whichever is appropriate.
  (3) Organize, where needed, meetings of the officers of the Subcommittees, using electronic forums, to discuss overarching matters.

### Article 6

#### Organization and functions of Subcommittees on Taxonomy and Ad Hoc Subcommittees

The formation of a Subcommittee on Taxonomy or an Ad Hoc Subcommittee may be proposed by an individual, a group of individuals or the EB-ICSP. A proposal must be accompanied by a description of the prokaryotic taxa or, alternatively, the overarching nomenclature-related topic to be studied, the reasons thereof and a list of proposed Subcommittee members. Such proposals shall be submitted to the Secretary for Subcommittees on Taxonomy for approval by the EB-ICSP. Subcommittees shall work under the following rules:

(a) Upon formation of a Subcommittee, the members will elect a Chair and Secretary and report their names to the Secretary for Subcommittees on Taxonomy. The terms of the officers of the subcommittees begin on 1 September 2020 and every 3 years thereafter. However, they may be re-elected, and there are no term limits. The functions of the Chair are to organize, with the Secretary, meetings of the Subcommittee, chair such meetings and coordinate all business as necessary with the ICSP and the Judicial Commission. The functions of the Secretary are to assist the Chair in organizing the Subcommittee meetings, prepare minutes of the meetings for publication [see Article 4(d)] and maintain a list of the members of the Subcommittee.
(b) A Subcommittee shall contain at least one regular member, which is affiliated with a Member Society of the BAM Division of the IUMS. The Subcommittee may co-opt other specialists. New members may be elected to a Subcommittee at any time. Prior to the election of officers [see Article 6(a)], the regular members of the Subcommittee shall decide, for the entire term, whether only regular members or also co-opted members of the Subcommittee shall be eligible for office and have the right to vote in the election of new members and officers.
  (1) The Secretary for Subcommittees on Taxonomy shall be a non-voting member of each Subcommittee and act as liaison between each Subcommittee, the EB-ICSP, the ICSP and the Judicial Commission.
  (2) Members who cannot attend meetings of a Subcommittee may designate an Alternate in a letter to the Subcommittee’s secretary. The designated Alternate may vote on the member’s behalf. However, no member or alternate of the subcommittee may cast more than one vote.
  (3) There is no term limit on membership in a Subcommittee, but all members should be actively engaged in the work of the Subcommittee. Members who cease to have interest in the work of the Subcommittee shall resign or be removed from the membership roster by a vote of the members of the Subcommittee.
  (4) Each Subcommittee is encouraged to meet at least once every year at any location considered appropriate by the Subcommittee’s members or hold electronic forums. Meetings and forums should be coordinated with the Secretary for Subcommittees on Taxonomy. A portion of the meeting may be restricted to regular members of the subcommittee or their alternates in order to deal, inter alia, with changes of membership and election of officers. Minutes of meetings will be communicated to the Secretary for Subcommittees on Taxonomy and published using appropriate means [see Article 4(d)].
  (5) Subcommittees on Taxonomy, which focus on particular taxa, shall work within the framework provided by the ICNP to:
    (a) Encourage and undertake research on the evolutionary relationships among the organisms in the taxa under study.
    (b) Evaluate and make recommendations regarding characters or procedures useful in distinguishing the organisms and taxa under study.
    (c) Make recommendations regarding classification of the organisms in the taxa under study. A Subcommittee on Taxonomy cannot legislate on classification but may contribute materially towards the general acceptance of a classification.
    (d) Make recommendations to the JC regarding nomenclature for the organisms in the taxa under study, including recommendations for the conservation or rejection of names.
    (e) Offer advice on recognition of the nomenclatural types of various taxa.
    (f) Recommend minimal standards for the description of new taxa. Such recommendations shall include a list of characters and procedures for their assessment and shall be reviewed at regular intervals. The standards shall be published in the IJSEM and/or other microbiological journals. Such standards shall be minimal and in no way limit the extent of investigation or contravene Principle 1(4) of the ICNP.
    (g) Maintain a list of validly published and other names that have been applied to the organisms in the taxa under study.
    (h) Include statements and recommendations relating to the above studies in Subcommittee minutes or submit them separately for publication in the IJSEM or elsewhere, as appropriate [Article 4(d)].
  (6) Ad Hoc Subcommittees, which focus on an overarching nomenclature-related topic, shall work within the framework provided by the ICNP to:
    (a) Evaluate and make recommendations regarding the overarching nomenclature-related topic that is their remit, as approved by the Executive Board.
    (b) Cooperate with other committees or similar bodies appointed to consider problems related to the overarching nomenclature-related topic.
    (c) Make recommendations regarding emendations of the ICNP.
    (d) Publish recommendations for addressing the overarching nomenclature-related topic in the IJSEM and/or other microbiological journals.
(c) **Dissolution of a Subcommittee on Taxonomy or an Ad Hoc Subcommittee.**
  (1) Subcommittees on Taxonomy and Ad Hoc Subcommittees may dissolve themselves if the members no longer consider that their work is required. The Secretary for Subcommittees on Taxonomy shall be informed.
  (2) Subcommittees that have not produced minutes over a 5-year period will be considered inactive and dissolved by action of the EB-ICSP if no Chair, Secretary or members can be identified to continue the work.
  (3) Ad Hoc Subcommittees will report regularly to the EB-ICSP via the Secretary for Subcommittees on Taxonomy and, if appropriate, using other means [see Article 4(d)] until completion of their remit, at which point a summary report will be prepared for submission to the ICSP and, if appropriate, publication in IJSEM or on other suitable platforms [see Article 4(d)].

### Article 7

#### The Judicial Commission

(a) **Functions of the Judicial Commission.** The Judicial Commission (JC) is a subsidiary of the ICSP and has the following functions:
  (1) To provide the ICSP expert advice on the interpretation of the ICNP, Requests for Opinions and other matters requested of it. To publish guidelines on the interpretation of the ICNP as deemed necessary.
  (2) To consider all Requests for Opinions relative to the interpretation of the ICNP (see Article 8). An action of the JC, such as the rejection or conservation of a name, shall only be issued when a majority of the Commissioners vote in favour. A tied vote is resolved by the vote of the three officers of the JC.
  (3) To consider proposals for emendation of the ICNP and make recommendations to the ICSP. As a minimum, this should include an assessment of possible unintended retroactivity, ambiguity and logical inconsistencies (internal and external) of the proposed changes.
  (4) To hold such sessions and electronic meetings as may be necessary to transact business.
  (5) To report to the EB-ICSP the names of all Commissioners whose terms of service will expire or any other vacancies in the membership of the Commission (e.g. due to resignation).
  (6) To cooperate with other commissions or similar bodies appointed to consider problems of nomenclature.
  (7) To consider enquiries from the wider scientific community on matters related to interpretation of the ICNP, particularly in regard to status of names as validly published or otherwise.
(b) **Organization.** The Judicial Commission (JC) shall consist of up to 12 and no less than 9 commissioners elected by the members of the ICSP. No member of the JC may serve as a voting member of the ICSP, and voting members of ICSP may not serve on the JC. This restriction is not retroactive.
  (1) Commissioners are elected by the members of the ICSP to serve in three classes of four or three Commissioners, one class retiring upon the election of a new class. A nomination for election requires the written support of at least one Full Member of the ICSP and should summarize the relevant expertise of the candidate. The terms of classes begin on 1 September 2020 and every 3 years thereafter. Members of retiring classes are eligible for re-election.
  (2) Immediately following the election of each new class of Commissioners, the Chair of the ICSP will organize the election of the officers of the JC from among its members. The officers will include the Chair, Vice-Chair and Secretary. The officers serve until 1 September 2020 and every 3 years thereafter but may be re-elected if their terms as commissioners have not expired.
  (3) In the case of resignation, removal from office or death of a Commissioner between elections, the vacancy is filled by ballot of the members of the ICSP. A Commissioner of one class is eligible for election to a vacancy occurring in another class but may only be a member of one class. Commissioners elected to fill a vacancy caused by resignation, removal from office or death shall serve for the unexpired term of the vacancy.
(c) **Duties of the Chair of the Judicial Commission**
  (1) To preside at meetings of the JC.
  (2) To prepare in collaboration with the Vice-Chair the agenda for meetings of the JC.
  (3) To participate in regular meetings of the EB-ICSP as a non-voting member to communicate relevant matters regarding the JC and ICSP [see Article 4(d)].
  (4) To serve on the Editorial Board for the ICNP (see Article 13).
  (5) To receive Requests for Opinion accepted for publication in the IJSEM from its Editor-in-Chief and to immediately transmit these to the JC for consideration.
  (6) To organize the JC’s consideration of Requests for Opinions (see Article 8) and recommendations regarding proposals for emendation of the ICNP [see Article 7(3)].
  (7) To represent the JC on such international committees, boards or commissions as may be organized to consider cooperation in biology on the solution of common problems of nomenclature and taxonomy, particularly to work with other similar commissions or executive committees organized for action on problems on nomenclature in other biological sciences.
  (8) To undertake such other duties as may from time to time be requested by the JC or the ICSP.
(d) **Duties of the Vice-Chair of the Judicial Commission**
  (1) To preside at meetings of the JC in the absence of the Chair. If both the Chair and the Vice-Chair are absent, the commissioners may elect an ad hoc Chair to preside at the meeting.
  (2) To assume all the duties of the Chair in the event of death or resignation of the Chair, until such time as a new Chair has been elected.
  (3) To prepare in collaboration with the Chair and the Secretary of JC the agenda for meetings of the JC.
  (4) To serve on the Editorial Board for the ICNP (see Article 13).
  (5) To participate in regular meetings of the EB-ICSP as a non-voting member to communicate relevant matters regarding the JC and ICSP [see Article 4(d)].
  (6) To undertake such other duties as may from time to time be requested by the JC or the ICSP.
**(e) Duties of the Secretary of the Judicial Commission**
  (1) To prepare and submit copies of the minutes of meetings of the JC for publication in the appropriate forum [see Article 4(d)].
  (2) To coordinate voting by members of the JC and to record the outcome of all votes.
  (3) To participate in regular meetings of the EB-ICSP as a non-voting member to communicate relevant matters regarding the JC and ICSP [see Article 4(d)].

### Article 8

#### Requests for Opinions

When necessary to resolve differences in opinion regarding the interpretation of the ICNP or when necessary to clarify other questions regarding the nomenclature of prokaryotes, the opinion of the JC may be requested.

(a) The first step in the Request for an Opinion is submission of a manuscript to the IJSEM requesting the opinion and providing the rationale.
(b) The Editor-in-Chief of the IJSEM will supervise the manuscript’s review by members of the JC and, if deemed necessary, by members of the relevant Subcommittee on Taxonomy, and/or ad hoc reviewers. IJSEM peer review does not antedate an Opinion of the JC but the manuscript should be rejected if it:
  (1) fails to adequately present its case within the framework and appropriate application of the ICNP
  (2) revisits a previously denied Request for an Opinion without presenting significant new information on the case.
  (3) requests the reconsideration of a previously issued Judicial Opinion but neither presents new evidence nor a new rationale. If the manuscript is accepted for publication, the Editor-in-Chief will ensure that the request is communicated to the Chair of the JC for consideration immediately upon acceptance (i.e. in preprint or ‘author accepted manuscript’ form).
(c) If the manuscript is rejected upon review, 7 voting members of the ICSP may request that the Request for an Opinion proceed further. In this case, the proposal is published in the IJSEM with a note identifying the members who support its publication.
(d) The proposal will then be processed by the JC within 6 months of publication in the IJSEM. If approved by a majority of the Commissioners, the Judicial Opinion shall be submitted to the IJSEM for publication. JC ballots should not have abstention as an option.
(e) Each Judicial Opinion must describe the reasons for granting or denying the Request for an Opinion, or parts thereof. The rationale presented therein must be related to the pertinent clauses of the ICNP and, if applicable, to previous publications of the JC. Commissioners who disagree with the majority vote on the Judicial Opinion may include a minority report alongside the main Judicial Opinion.
(f) Decisions of the Judicial Commission may be overturned in a subsequent Judicial Opinion.
(g) In the absence of the submission of an Opinion for publication by the JC within half a year of the publication of the Request for an Opinion, the Executive Secretary of the ICSP shall reprimand the JC for the delay. In the absence of the submission of an Opinion for publication by the JC within one year of the publication of the Request for an Opinion, the Executive Secretary of the ICSP shall initiate an investigation by the EB-ICSP into the removal of members of the JC from office (see Article 17).

### Article 9

#### Publications Committee

(a) The members of the Publications Committee shall be the Chair and Vice-Chair of the ICSP, the Chair and Vice-Chair of the JC, the Executive Secretary of the ICSP, the Secretary for Subcommittees on Taxonomy and the Editor-in-Chiefs of the publications authorized by the ICSP. The Publisher of the IJSEM is entitled to send an ex officio delegate to the Publications Committee.
  (1) The Vice-Chair of the ICSP shall be Chair of the Publications Committee.
  (2) The Executive Secretary shall be Secretary of the Publications Committee.
  (3) Should a conflict of interest arise for any member of the Publications Committee, an alternate will be appointed from full members of the ICSP by the remaining members of the Committee.
(b) The functions of the Publications Committee shall be the following:
  (1) Make recommendations to the ICSP for the establishment of such publications as deemed necessary for the advancement of the systematics of prokaryotes and their Editorial Boards.
  (2) Be responsible for the publication of the *International Journal of Systematics and Evolutionary Microbiology* (IJSEM), *International Code of Nomenclature of Prokaryotes* (ICNP), the *Approved Lists of Bacterial Names* and the ‘Statutes of the International Committee on Systematics of Prokaryotes’.
  (3) To receive an annual report, including a financial statement from audited accounts, from the Publisher regarding the publication of IJSEM and to report on this to the EB-ICSP.
  (4) Submit a report of the Publications Committee through the Executive Secretary of the ICSP for transmission to ICSP at each plenary meeting.

### Article 10

The official publications of the ICSP are the IJSEM (formerly *International Journal of Systematic Bacteriology*), ICNP (formerly *International Code of Nomenclature of Bacteria*), ‘Statutes of the International Committee on Systematics of Prokaryotes’ and *Approved Lists of Bacterial Names*.

### Article 11

**Sponsorship of Publications.** No publication of the ICSP or any of its organizations shall bear any indication of sponsorship by a commercial company, or institution connected in any way with a commercial company, except an acceptable acknowledgement of financial assistance. In cases of doubt, the EB-ICSP will make the final decision.

### Article 12

**Editorial Boards.** Editorial Boards shall be proposed by the EB-ICSP. Currently, two Editorial Boards are recognized:

(a) The Editorial Board for the ***International Code of Nomenclature of Prokaryotes*** **(ICNP)**.
(b) The Editorial Board for the ***International Journal of Systematic and Evolutionary Microbiology* (IJSEM)**.

### Article 13

The Editorial Board for the *International Code of Nomenclature of Prokaryotes* (ICNP) shall consist of an Editor-in-Chief appointed by the EB-ICSP, the Chair and the Vice-Chair of the JC and the Chair and Vice-Chair of the ICSP. The Editor-in-Chief shall be the Chair of the Editorial Board. The Board shall have power to co-opt.

(a) **Duties of Editor-in-Chief of the ICNP.** The Editor-in-Chief shall be responsible for the continuing revision of the ICNP, for its editing and its publication. The Editor-in-Chief shall submit the manuscript after approval by the Editorial Board for publication in book form and/or electronic form.
  (1) To supervise consideration of proposals for emendation by the ICSP after publication in the IJSEM, including distribution of comments from the JC and members of the ICSP to the entire ICSP, and supervision of balloting among the ICSP.
(b) **Emendation of the ICNP.** The emendation of the Code shall follow this procedure:
  (1) The first step in the emendation of the ICNP is submission of a manuscript to the IJSEM requesting emendation and providing the rationale.
  (2) The Editor-in-Chief of the IJSEM will supervise the manuscript’s review by the ICNP Editorial Board and/or ad hoc reviewers. If the manuscript is recommended for publication, the Editor-in-Chief of the IJSEM will ensure that the request for emendation is communicated to the Editor-in-Chief of the ICNP, who will supervise its further consideration by the ICSP and JC.
  (3) If the manuscript is rejected upon review, seven voting members of the ICSP may request that the proposal for emendation proceed further. In this case, the proposal is published in the IJSEM with a note identifying the members of the ICSP supporting it.
  (4) The members of the ICSP, the JC and other interested parties (including the authors of the proposal) will have 3 months from the publication in the IJSEM to provide comments to the Editor-in-Chief of the ICNP. The EB-ICSP will decide on the nature of any accompanying public discussion deemed appropriate. The authors of the proposal will then have up to 6 weeks to respond to the comments. Based on the comments, the authors may present a revised version for approval in place of the published version but must retain the quintessence of the proposal and explain the changes made. After that time, the proposal and all comments will be communicated to the members of the ICSP for voting.
  (5) The ICSP will make its decision within 6 weeks. A summary of the debate and the outcome of the vote will be published in the IJSEM. Emendations take effect from the day of publication.
  (6) If a proposal is not accepted, the authors or other parties may request the voting members of the ICSP to reconsider its decision by submitting a corresponding manuscript to the IJSEM [see Article 13(b)(1)]. This manuscript should present new evidence and/or a new rationale for supporting the emendation of the ICNP and/or propose an adapted phrasing that addresses critical comments on the previous proposal(s).

### Article 14

**Editorial Board for the *International Journal of Systematic and Evolutionary Microbiology***. The Editorial Board shall consist of an Editor-in-Chief appointed by the EB-ICSP, the Vice-Chair of the ICSP, a representative appointed by the Publisher, and the Editors that are appointed as described below. The Editor-in-Chief will be Chair of the Editorial Board and may not serve concurrently as the Chair or Vice-Chair of the ICSP.

(a) The functions of the Editorial Board shall include implementation of the ICNP, including the General Considerations, Principles, Rules, Recommendations, and the appropriate aspects of the Appendices, within the IJSEM.
(b) The Editorial Board shall also implement recommendations made to it by the ICSP, JC or appropriate Ad Hoc Subcommittees formed by the ICSP to deal with specific matters relating to publications that appear in the journal.
(c) Nominations for the Editorial Board should be sent to the Editor-in-Chief for consideration. The Editor-in-Chief appoints the Editors subject to the approval of the EB-ICSP. If considered necessary, e.g. if an Editor does not perform the functions in a satisfactory way, the Editor-in-Chief may remove an Editor subject to the approval of the EB-ICSP.
(d) The Editor-in-Chief shall be responsible for the review, acceptance and rejection of submitted manuscripts and for the supervision of the Editors.
(e) The Editor-in-Chief and the Editors shall be appointed for terms of up to 5 years, subject to reappointment and earlier termination. Editors may serve for no more than two consecutive terms in any one capacity.
(f) Policy matters deemed important by the ICSP shall be communicated through the EB-ICSP to the Editor-in-Chief. Policy matters initiated by the Publisher shall be communicated through the Editor-in-Chief to the EB-ICSP.
(g) Editorial policy, whether initiated by the ICSP, the Publisher or elsewhere, shall be communicated through the Editor-in-Chief to the Editors.
(h) The Editor-in-Chief may serve on the Publisher’s editorial policy committee representing the ICSP. The Editor-in-Chief shall act on behalf of the EB-ICSP, except in respect to the negotiation of contracts with the Publisher. In other transactions with the Publisher, the Editor-in-Chief shall forward copies of correspondence immediately to members of the EB-ICSP.
(i) The Publisher will submit all material for publication in the IJSEM to the Editor-in-Chief and Editors. The Editor-in-Chief and Editors shall have the power to refer such submissions to referees for assessment and reject such submissions that are deemed unsuitable for publication, provided that, in such instances, authors shall have the right of appeal via the Chair of the Editorial Board.
(j) The EB-ICSP shall be entitled to receive annually such part of the management accounts of the Publisher as relate to publication of the IJSEM and the audited accounts of the Publisher and to have access to such part of the books and records of the Publisher as are relevant to publication of the IJSEM at any time on giving reasonable notice and during normal business hours.
(k) Minutes of the Editorial Board meeting will be made available to the EB-ICSP.

### Article 15

No one connected with a commercial firm may use his or her connection with the ICSP or any of its committees, commissions and organizations, either as a member or officer, to advertise or promote his or her commercial interests in any way. Members of the EB-ICSP, JC, Editor-in-Chief and Editors of the IJSEM and ICNP shall submit a conflict of interest statement to the Chair of the ICSP upon the time of their appointment and must notify the Chair of any changes in their conflicts of interest during their terms of office.

### Article 16

#### Amendments of the Statutes

(a) Five or more voting members of the ICSP may initiate amendment of the statutes by submission of a manuscript to the IJSEM requesting amendment and providing the rationale.
(b) As deemed necessary, the Editor-in-Chief of the IJSEM will supervise the manuscript’s review by members of the EB-ICSP and/or ad hoc reviewers. Following the response of the authors to the reviewers’ comments and submission of revisions if desired, the Editor-in-Chief will ensure timely publication in IJSEM and communication to the Executive Secretary of the ICSP for consideration by the membership.
(c) The proposal will then be debated by the ICSP within 3 months of publication in the IJSEM, and all comments compiled. The authors of the proposal will then have up to 6 weeks to respond to the comments. Based on the comments, the authors of the proposal may present a revised version for approval in place of the published version but must retain the essence of the proposal and explain the changes made. After that time, the proposal and all comments will be communicated to the Full Members of the ICSP for voting.
(d) The ICSP will make its decision within 6 weeks. Approval will require a vote of a majority of the members eligible to vote [see Article 2(e)]. Notice of amendments approved by the ICSP shall be published in the IJSEM as minutes of meetings of the ICSP or EB-ICSP.

### Article 17

Officers of the ICSP, including members of the Executive Board, the Judicial Commission, and the Subcommittees of Taxonomy and Ad Hoc Subcommittees, may be removed from office by a vote of the Full Members of the ICSP. Reasons for removal from office include failure to perform the responsibilities of the office, using the office for purposes other than those specified in the Statutes, violation of the conflict-of-interest statement, and acting in any other way that significantly contravenes the Statutes.

(a) Removal from office is initiated by a letter from five or more Full Members to the Executive Secretary or, if the Secretary is the subject of the complaint, to the Chair of the ICSP. The letter should specify the basis for removal. The officer will then have 6 weeks to respond. At the end of that time, the proposal to remove and the officer’s response will be circulated to the members of the ICSP for a consideration. Decisions to remove an officer require a vote by a majority (i.e. more than 50%) of the members eligible to vote and shall be reached on the basis of a qualified majority of two-thirds of those members who vote.
(b) A Full Member [Article 2(a)] may be removed on the same grounds as for the removal of officers by the Member Society which appointed the Full Member. A request for removal shall be notified to the Society by the EB-ICSP. If necessary, the matter may be referred to the IUMS Executive Board.
(c) Co-opted Members [Article 2(b)] and Life Members [Article 2(c)] may be removed from membership on the same grounds and by the same procedure as for the removal of officers.

## Abbreviations

BAM: Bacteriology and Applied Microbiology Division of IUMS
EB: Executive Board
ICNP: International Code of Nomenclature of Prokaryotes
ICSP: International Committee on Systematics of Prokaryotes
IJSEM: International Journal of Systematic and Evolutionary Microbiology
IUMS: International Union of Microbiological Societies
JC: Judicial Commission
